# Utility of a Telephone Triage Hotline in Response to the COVID-19 Pandemic: Longitudinal Observational Study

**DOI:** 10.2196/28105

**Published:** 2021-11-01

**Authors:** Benjamin A Y Cher, Eric A Wilson, Alexa M Pinsky, Ryan F Townshend, Ann V Wolski, Michael Broderick, Allison M Milen, Audrey Lau, Amrit Singh, Sandro K Cinti, Carl G Engelke, Anjan K Saha

**Affiliations:** 1 Michigan Medicine University of Michigan Ann Arbor, MI United States; 2 Department of Internal Medicine Michigan Medicine University of Michigan Ann Arbor, MI United States; 3 Department of Anesthesiology Columbia University Irving Medical Center New York, NY United States

**Keywords:** triage, telephone, COVID-19, utility, telemedicine, telehealth, patient information, concern, implementation, innovation, hospital

## Abstract

**Background:**

During the initial months of the COVID-19 pandemic, rapidly rising disease prevalence in the United States created a demand for patient-facing information exchanges that addressed questions and concerns about the disease. One approach to managing increased patient volumes during a pandemic involves the implementation of telephone-based triage systems. During a pandemic, telephone triage hotlines can be employed in innovative ways to conserve medical resources and offer useful population-level data about disease symptomatology and risk factor profiles.

**Objective:**

The aim of this study is to describe and evaluate the COVID-19 telephone triage hotline used by a large academic medical center in the midwestern United States.

**Methods:**

Michigan Medicine established a telephone hotline to triage inbound patient calls related to COVID-19. For calls received between March 24, 2020, and May 5, 2020, we described total call volume, data reported by callers including COVID-19 risk factors and symptomatology, and distribution of callers to triage algorithm endpoints. We also described symptomatology reported by callers who were directed to the institutional patient portal (online medical visit questionnaire).

**Results:**

A total of 3929 calls (average 91 calls per day) were received by the call center during the study period. The maximum total number of daily calls peaked at 211 on March 24, 2020. Call volumes were the highest from 6 AM to 11 AM and during evening hours. Callers were most often directed to the online patient portal (1654/3929, 42%), nursing hotlines (1338/3929, 34%), or employee health services (709/3929, 18%). Cough (126/370 of callers, 34%), shortness of breath (101/370, 27%), upper respiratory infection (28/111, 25%), and fever (89/370, 24%) were the most commonly reported symptoms. Immunocompromised state (23/370, 6%) and age >65 years (18/370, 5%) were the most commonly reported risk factors.

**Conclusions:**

The triage algorithm successfully diverted low-risk patients to suitable algorithm endpoints, while directing high-risk patients onward for immediate assessment. Data collected from hotline calls also enhanced knowledge of symptoms and risk factors that typified community members, demonstrating that pandemic hotlines can aid in the clinical characterization of novel diseases.

## Introduction

COVID-19 emerged in December 2019 as a highly contagious viral pneumonia caused by SARS-CoV-2 [1]. The disease is transmitted through aerosolized respiratory droplets and was classified as a pandemic by the World Health Organization on March 11, 2020, after rapidly crossing intercontinental and transoceanic boundaries over three months [2]. Symptoms include fever, cough, and dyspnea; unmanaged disease can result in multiorgan failure and possibly death [3,4]. The global response to COVID-19 has focused on medically managing the disease, optimizing hospital utilization, and reducing viral transmission [5].

The rising prevalence of COVID-19 in the United States [6] created a demand for patient-facing information exchanges that addressed questions and concerns about the disease. Local efforts designed to contextualize national facts and figures were critical for layering population-specific considerations over nationally accepted guidelines. As the disease began to spread, acute care settings experienced a surge in patients seeking treatment and advice, stressing already overburdened health care systems and creating infection control challenges in overcrowded waiting rooms [7,8]. Methods aimed at managing this sudden spike in health care utilization required adherence to social distancing recommendations to prevent viral transmission while simultaneously alleviating the added systemic congestion attributed to COVID-19.

One approach to managing increased patient volumes during a pandemic involves the implementation of telephone-based triage systems. Triage and evaluation phone lines established during previous pandemics were found to be cost-effective methods for prioritizing acute care resources for symptomatic patients with risk factors for serious disease in overburdened hospital systems [9-11]. A study of infectious disease hotlines implemented following natural disasters showed that these systems effectively aid patient evaluation and prevent disease outbreaks in vulnerable populations [12]. Furthermore, disease triage hotlines serve as effective information waypoints for collecting longitudinal patient data while relieving congestion that would otherwise exacerbate disease propagation [13]. However, a lack of consensus on a universal approach to triage due to variation in disease presentation across institutions and geographic areas prevents scalable implementation of triage hotlines [14-16]. Approaches that optimize resource allocation by accounting for variation in disease presentation thus present compelling value propositions for health systems worldwide to address ongoing and future pandemics.

To this end, we aimed to evaluate the COVID-19 triage hotline established by Michigan Medicine, the academic medical center affiliated with the University of Michigan, which serves the population of Southeast Michigan and surrounding areas. We characterized the call center’s triage algorithm and described the distribution of all calls fielded over a 6-week interval during the initial surge of the pandemic. We then contextualized hourly and daily call volumes against publicly available internet search trends concerning COVID-19–related inquiries. Lastly, we tabulated symptomatology and risk factor profiles for patients who accessed call center services during the 6-week period to provide perspective on local disease burden. Our results illustrate the utility of telephone-based triage and evaluation hotlines in optimizing health care utilization and surveying disease burden during times of crisis that impact human health.

## Methods

### COVID-19 Call Center Hotline and Triage Algorithm

Michigan Medicine established a telephone hotline to triage inbound patient calls related to COVID-19. Operations commenced on March 16, 2020, and accepted calls daily from 6 AM to 12 AM. The hotline was staffed both in person and remotely by call center agents, medical assistants, patient service representatives, and medical student volunteers (herein termed as “agents”). The call center used Aspect Call Center software (Aspect) to receive and manage calls. Call center agents used an algorithm developed by the Michigan Medicine Ambulatory Care Administration to triage incoming calls with screening questions and defined endpoints. Screening questions were updated over the duration of the study to accommodate the changing health landscape of the pandemic. Symptom screening criteria were initially limited to fever, cough, and dyspnea. Criteria were expanded to include upper respiratory symptoms (runny nose and congestion), muscle aches, loss of sense of smell, and diarrhea on April 20, 2020. A positive symptom screen was defined as having at least two symptoms from this list. Risk factor criteria initially included age over 65 years, compromised immune system, chronic respiratory illness, presence of a health care worker in the household, hemodialysis patient, resident of a skilled nursing or long-term care facility, and/or living in communal housing. Risk factor criteria were expanded to include primary caretaker for a vulnerable individual and/or close contact of an individual who tested positive for COVID-19 on April 20, 2020. A positive risk factor screen was defined as having at least one risk factor from this list.

### Data Sources

We used two surveys to collect data for this analysis. The first was created in Qualtrics, an electronic survey platform. Call center workers filled out a Qualtrics survey when they handled each call to the call center. From these results, we extracted call volume, patient employment status (Michigan Medicine—and later, University of Michigan—employee versus nonemployee), patient status (Michigan Medicine versus non–Michigan Medicine patient), pediatric or adult, symptom profile, and risk factor data**.** The triage survey directed callers toward various algorithm endpoints: Occupational Health Services, non–Michigan Medicine primary care provider or the Centers for Disease Control and Prevention website, patient portal e-visit questionnaire, and adult or pediatric nursing hotlines. Partially completed Qualtrics survey responses were discarded from downstream analysis. The second survey was completed by a subset of call center workers who were volunteers during evening shifts. This survey was created in Google Forms, an additional online survey platform. The Google Forms survey collected additional data about symptomatology and risk factors that were not included in the Qualtrics survey. Qualtrics and Google Forms survey data were merged to produce a composite data set at the end of the study period. We used an additional data source, the Aspect Unified IP System, which automatically logged all telephone calls to our institution’s call center hotline. We used this source to cross-check data on call volumes extracted from the Qualtrics and Google surveys.

To contextualize how call volume was related to public interest in the pandemic and disease prevalence, we accessed publicly available data through two sources: (1) Google Trends and (2) the State of Michigan online coronavirus dashboard [17]. We compiled regional trends in searches for the terms “COVID,” “COVID-19,” and “Coronavirus” during the study period. We also compiled search trends for words that mirrored the symptoms in our screening algorithm: “Fever,” “Cough,” “Short of Breath,” “Shortness of Breath,” and “Trouble Breathing.” Data provided by Google Trends are normalized to a maximum of 100 for the frequency of searches between March 24, 2020, and May 5, 2020, for the state of Michigan and the city of Detroit. Data provided by the State of Michigan online coronavirus dashboard were presented as 3-day total confirmed cases and deaths from COVID-19 in Washtenaw county during the same time period.

In tandem with the creation of a call center to assess patients, Michigan Medicine created a “Cough, Flu, and COVID-19–like Symptoms” patient portal e-visit questionnaire. Patients calling the triage hotline whose symptoms or risk factor profile did not meet criteria to be transferred to the nursing line were directed to complete this e-visit questionnaire in their patient portal. The questionnaire assessed symptom frequencies, symptom onset and duration, and epidemiologic and demographic risk factors. Once completed, a provider would reach out to the patient to determine appropriate next steps in care.

### Data Analysis

Data were collected between March 24, 2020 (8 days after the triage hotline began operation) and May 5, 2020. Total call volume and the total number of calls directed to each triage algorithm endpoint by date and time of day were calculated to assess hotline workload. Symptom frequency was subsequently overlaid on total call volume for the call center and the total number of calls in which patients screened positive for symptoms. Google Forms survey data and the “Cough, Flu, and COVID-19–like Symptoms” patient portal questionnaire responses were analyzed to determine the frequency of patient-reported symptoms and epidemiologic risk factors among both hotline callers and online portal users. Data representation and statistical analysis was completed using Prism (version 8.4.2; GraphPad). The study was reviewed and approved by the local institutional review board (HUM00179879).

## Results

### Call Volumes and Algorithm Endpoints

**Figure 1:**
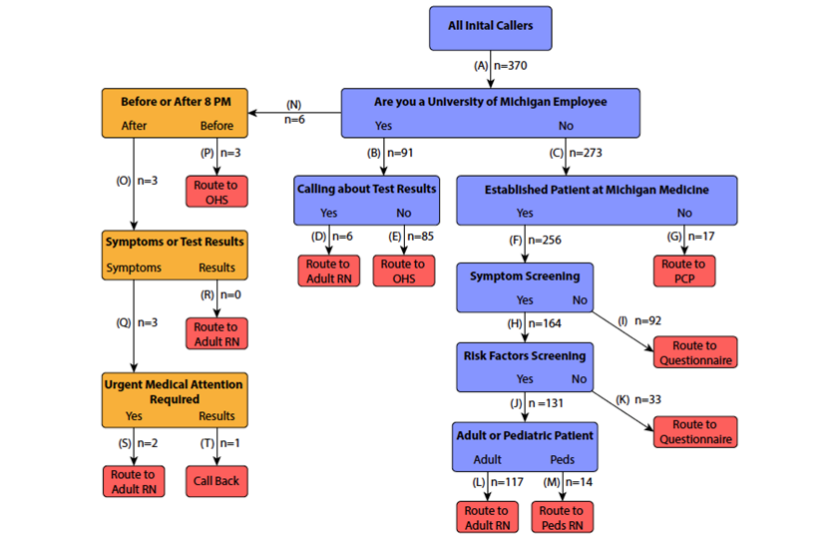
Detailed workflow of the Michigan Medicine COVID-19 Hotline. Outline of triage algorithm, including initial input/questions (blue), endpoints (red), and an alternative pathway added on May 1, 2020 (orange). The numbers of callers routed through each pathway are shown at unique junctions in the algorithm. OHS: Occupational Health Services; PCP: primary care provider; RN: registered nurse.

Upon establishing a staffed triage hotline, calls were processed through the screening algorithm, which directed patients to specific algorithm endpoints (Figure 1).

**Figure 2:**
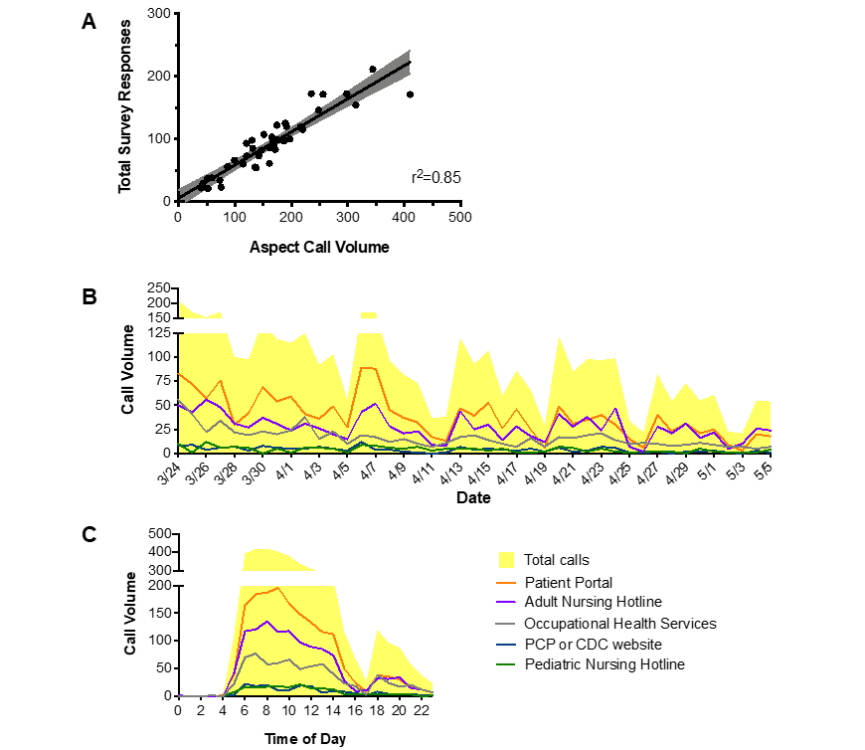
Linear regression analysis of survey responses versus number of incoming calls and daily/hourly call volumes. (A) The number of unique Qualtrics and Google Forms survey responses collected during the study period had a strong positive correlation with COVID-19 Hotline call volumes recorded by Aspect software during the same time frame (Pearson coefficient, *r*^2^=0.85). (B) Daily and (C) hourly call volumes obtained from combined Qualtrics and Google Forms survey data, with total calls received graphed as a stacked line plot (yellow) with subcategories of triage algorithm endpoints overlaid as line graphs. Orange: routed to patient portal; purple: routed to adult nursing; grey: routed to OHS; blue: routed to PCP or Centers for Disease Control and Prevention website; green: routed to pediatric nursing. OHS: Occupational Health Services; PCP: primary care provider.

To establish that our survey data provided an accurate representation of our call volume, we compared daily call volume to the number of calls routed to the hotline number through Aspect Unified IP, which automatically logged all calls to the call center hotline (Figure 2A). Although the Google and Qualtrics survey response rate was approximately 50% of Aspect system forwarding, the linear relationship between call volume as measured by the surveys and the Aspect system (*r*^2^=0.85) suggests that survey responses provide a reasonable representation of call volume received by the triage hotline.

A total of 3929 calls (average 91 calls per day) were received by the call center during the study period. Overall, call volume gradually declined over time but showed substantial variation between days throughout the study period (Figure 2B). The distribution of calls between algorithm endpoints was consistent through the study period. Callers were most often directed to the institutional patient portal (1654/3929, 42%), nursing hotlines (1338/3929, 34%), or Occupational Health Services (709/3929, 18%). Of the 1338 calls directed to the nursing hotline, 1164 (1164/1338, 87%) were forwarded to the adult nursing hotline and 174 (174/1338, 13%) were sent to the pediatric nursing hotline. The total number of calls peaked at 211/day on March 24, 2020, and fell to 54/day by May 5, 2020, when data collection ended. Incoming call volumes were highest between 6 AM and 11 AM and steadily decreased throughout the remainder of the day with the exception of a brief resurgence between 6 PM and 12 AM (Figure 2C). Additionally, hourly call volumes were lower on the weekends than during weekdays, but volumes were similarly distributed throughout the day (Figure S1 in Multimedia Appendix 1).

**Figure 3:**
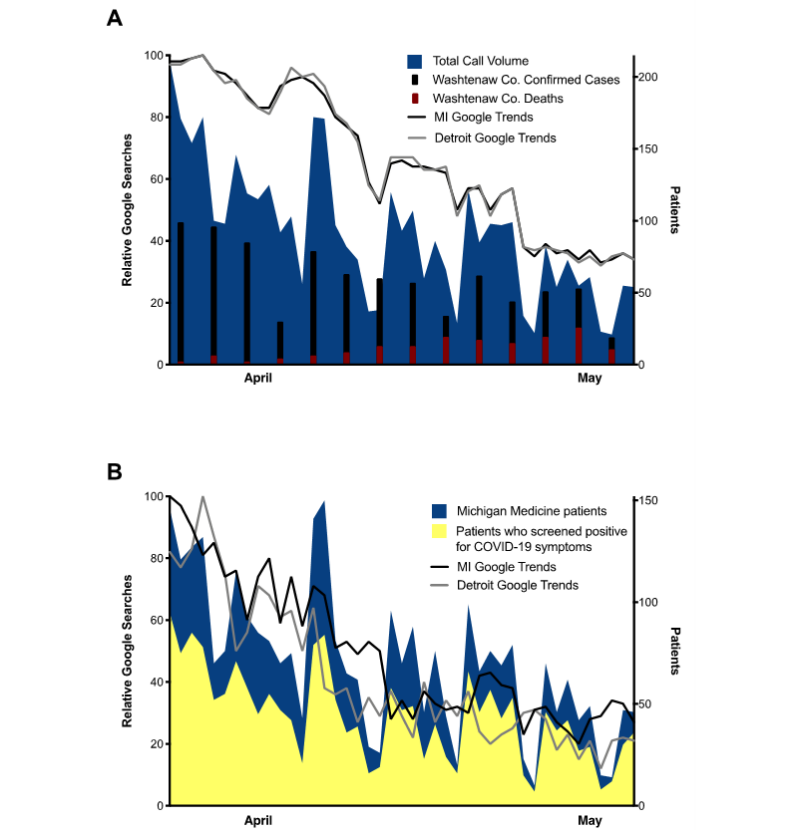
Google Search and hotline call volume trends. (A) Total call volume derived from combined Qualtrics and Google Forms survey data graphed as a stacked line plot (blue), relative frequencies of COVID-19–related Google Search results for the state of Michigan (MI, black) and Detroit area (grey) overlaid as line plots, and 3-day total confirmed cases (black) and deaths (red) from COVID-19 in Washtenaw county represented as bars. (B) Total number of callers who were Michigan Medicine patients (blue stacked line plot) and those who screened positive for symptoms (yellow stacked line plot) overlaid with relative frequencies of Google Search results pertaining to COVID-19 symptoms in the state of Michigan (black) and Detroit area (grey) overlaid as line plots. For panels (A) and (B), the left y-axis represents the normalized range (0-100) of Google Trends results, and call volumes and patient numbers are represented on the right y-axis.

We were interested to see whether call volume and the fraction of callers experiencing symptoms was reflective of the public interest in the pandemic and locoregional case volume. Google Trends frequencies for COVID-19–related searches for the state of Michigan and the Detroit area were highest in late March and gradually decreased until the end of data collection in early May (Figure 3A). Confirmed cases for Washtenaw County and total triage hotline call volume followed a similar overall trend, though both demonstrated lulls followed by sudden spikes in early and mid-April. Searches related to the most common symptoms of COVID-19 in both Michigan and Detroit peaked in late March and early April, mirroring the peak in call volume and callers screening positive for symptoms (Figure 3B).

### Caller Symptomatology and Risk Factors

**Figure 4:**
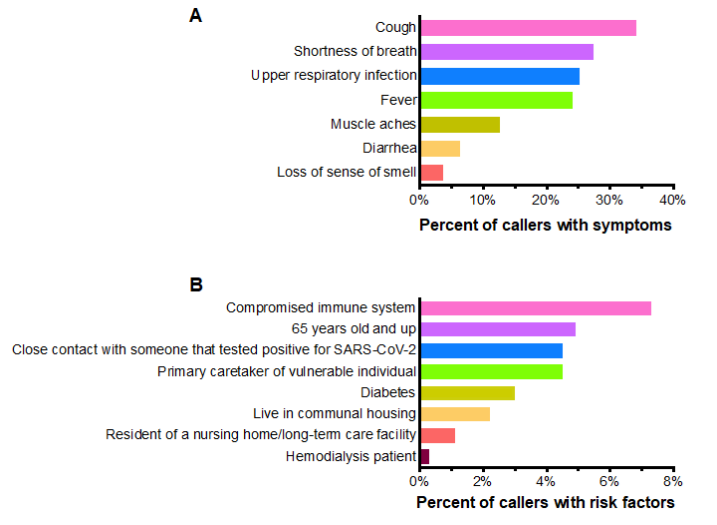
Symptoms and risk factors reported by hotline callers. Percent of patient-reported (A) symptoms and (B) risk factors obtained from Google Forms survey data. For "Cough," "Shortness of breath," "Fever," "Compromised immune system," "65 years old and up," "Hemodialysis patient," "Diabetes," "Resident of a nursing home/long-term care facility," and "Living in communal housing," n=370, while for "Upper respiratory infection," "Muscle aches," "Diarrhea," "Loss of sense of smell," "Close contact with someone that tested positive for SARS-CoV-2," and "Primary caretaker of a vulnerable individual," n=111, as these were added to the screening algorithm at a later study timepoint.

We next analyzed Google Forms survey data to determine the frequency of patient-reported symptoms and the distribution of epidemiologic risk factors among callers. These represent the subset of callers who were Michigan Medicine patients and contacted the call center between 6 PM and 12 AM. Cough (126/370, 34% of callers), shortness of breath (101/370, 27%), upper respiratory infection (28/111, 25%), and fever (89/370, 24%) were the most commonly reported symptoms (Figure 4A). A minority of callers reported muscle aches, diarrhea, or loss of sense of smell (14/111, 13%; 7/111, 6%; and 4/111, 4%, respectively). Among the risk factors assessed, immunocompromised state (23/370, 7%), age >65 years (18/370, 5%), primary caretaker of a vulnerable individual (5/370, 4.5%), and close contact with a person known to have tested positive for SARS-CoV-2 (5/370, 4.5%) were the most frequently reported by hotline users (Figure 4B).

### Patient Portal Symptomatology and Risk Factors

**Figure 5:**
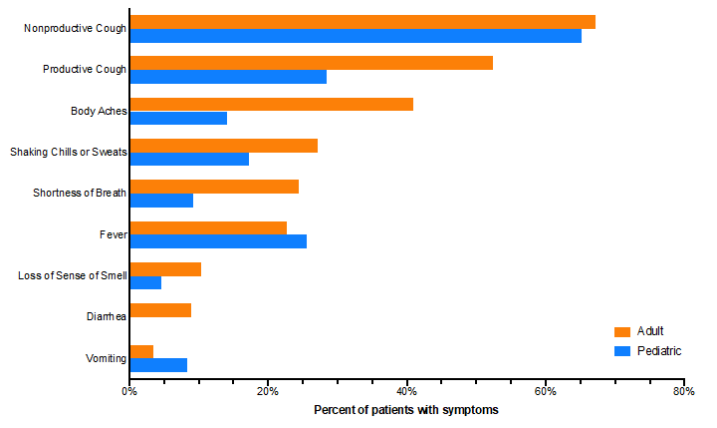
"Cough, Flu, and COVID-19–like Symptoms" e-visit questionnaire symptom profiles for adult and pediatric patients. (A) Percent of reported symptoms among adult (orange, n=1907) and pediatric (blue, n=227) patients who completed the e-visit questionnaire within the study time frame.

Further characterization of patient-reported symptoms and epidemiologic risk factors was achieved through “Cough, Flu, and COVID-19–like Symptoms” questionnaire responses. Questionnaire data revealed that nonproductive or productive cough were the most frequently reported symptoms among both adult (nonproductive: 1280/1907, 67%; productive: 998/1907, 52%) and pediatric (nonproductive: 148/227, 65%; productive: 64/227, 28%) patients (Figure 5). Muscle aches were the next most common symptom reported by adults (780/1907, 41%), while pediatric patients were more likely to experience fever (58/227, 26%). Adult patients were more likely to experience dyspnea than pediatric patients (465/1907, 24% versus 21/227, 9%) and loss of sense of smell (198/1907, 10% versus 10/227, 5%). Gastrointestinal symptoms (vomiting, diarrhea) were reported by <10% of adult and pediatric patients. Regarding clinical time course, both adult and pediatric patients were more likely to report a slowly progressive disease course (67%, 1393/2081 of adults and 61%, 118/194 of pediatric patients) than acute onset of illness (33%, 688/2081 of adults and 39%, 76/194 of pediatric patients). Health care worker (12%, 259/2132 of adults) and household contact of a health care worker (15%, 30/196 of pediatric patients) were the most frequently reported risk factors (Figure S2A,B in Multimedia Appendix 1). Of adult patients, 7% (142/2132) indicated that they had a compromised immune system compared to 2% (4/196) of pediatric patients.

## Discussion

### Principal Findings

In this study, we illustrated an approach to rapid development and implementation of a telephone-based triage system during the COVID-19 pandemic. Although call volumes fluctuated considerably throughout the study period, we observed a gradual decline in total calls received over time. We noted that referral to the patient portal or transfer to the nursing hotline for further assessment constituted more than 75% of triage decisions. Google Trends data for COVID-19–related terminology and symptoms in Michigan and the Detroit metropolitan area decreased from late March to early May 2020, mirroring the declining trend in call volumes noted at the call center. Callers most frequently reported the following symptoms: cough, shortness of breath, upper respiratory infection, and fever. In addition, the following risk factors for infection were most frequently reported: immunocompromised, age greater than 65 years, and exposure to another individual who tested positive for COVID-19. Analysis of patient questionnaire data revealed that cough was the most frequently reported symptom by adult and pediatric patients, but the frequency of symptoms thereafter differed between these groups. Together, these results demonstrate how patient hotlines and triage systems can be used to properly allocate care and broaden our understanding of disease characteristics during public health crises.

Responding effectively to a global pandemic necessitates judicious use of resources and regular assessment of disease burden. Tools with this dual capability are positioned to meaningfully contribute to pandemic response efforts. Specifically, triage and evaluation phone services are well-equipped to manage and survey patient populations when deployed by health systems during pandemics. Characterization of the Michigan Medicine COVID-19 Hotline validated this notion. The call center successfully directed call traffic away from the resource-intensive nursing endpoints while capturing population-level statistics on disease symptomatology and prevalence. This was accomplished by direct triage and surveillance by call center agents as well as questionnaire-based surveillance through a pre-existing online portal. Only 17% of the total call volume was directed to the nursing hotlines, while 21% of calls were directed to the online patient portal. Cough was identified as the most common symptom by triage agents as well as questionnaires, coinciding well with prior reports on COVID-19 symptomatology [1,5]. Taken together, the Michigan Medicine COVID-19 Hotline effectively triaged patients seeking advice and care during the COVID-19 pandemic while facilitating characterization of local disease burden.

Prior triage hotlines have provided population-level statistics about disease symptomatology and prevalence while directing resource allocation [13]. During the H1N1 pandemic of 2009, triage lines were developed to facilitate rapid diversion of call traffic from upper-level providers [9,11,13] while providing reassurance to patients with less concerning symptoms [11]. In addition, total call volume may itself be a useful marker for informing appropriate resource management. The tight concordance observed between regional Google Trends search term frequencies and total call volume is in line with the findings of previous studies comparing search trends to disease incidence [18,19] and supports the notion that call center traffic can operate as a barometer for community concerns surrounding COVID-19.

Designing the triage algorithm to leverage an online patient portal not only reduced nursing utilization but also unlocked local care capacity without compromising social distancing measures designed to reduce viral transmission. Previous work has demonstrated that call centers can also benefit a community beyond their primary role as a triage service. For instance, South Australia’s COVID-19 Relief Call Centre assisted callers with food insecurity and accessing medical appointments and welfare checks [20]. Call centers additionally provide alternative employment opportunities during pandemic shutdowns for both health care workers and non–health care workers. As an example, nurses and medical assistants on furlough at Michigan Medicine were redeployed as call center agents. In New York City, customer service agents at a travel management firm were trained and deployed as COVID-19 test schedulers [21]. Call centers have also been an effective means of reaching underserved patients in rural areas [22].

Nevertheless, a number of operational challenges place constraints on the value of call centers. Hotline staff do not perform physical exams, which are often necessary to properly evaluate the cardiopulmonary complaints that are characteristic of COVID-19. Maintaining social distancing standards and properly sanitizing shared workspaces can also pose a challenge to call center operations during pandemics [23]. To circumvent these challenges, hotlines based on yes/no algorithms can be supplemented by online self-triage systems [24]. Social distancing practices can be maintained by adopting work from home strategies, as was done by a call center in England that transitioned almost 1000 employees to remote work at the outset of the pandemic [25]. Although imbalances between health care supply and demand are likely to arise during future pandemics or subsequent waves of the COVID-19 pandemic, call center hotlines can be used to reduce the burden placed on strained medical systems.

### Limitations

A number of operational constraints placed limitations on the scope of our study. First, the Google Forms survey designed to screen symptomatology and risk factors was only completed for evening callers. Specific symptoms or risk factors associated with the time frame in which calls were received may have confounded our results. Second, due to the structure of the triage algorithm, we were only able to present symptomatology and risk factor data for a small subset of patients. Further, since the triage algorithm screened callers as positive if they had two symptoms or one risk factor, it is possible that additional symptoms or risk factors were not captured once a caller had screened positive. Third, not all callers had questions or requests that fit perfectly into the triage algorithm. For these calls, call center workers may have skipped the online survey (leading to inaccurate measurements of call volume) or filled out the survey with their best approximation of the caller’s needs. Fourth, due to the deidentified nature of our data sets, we were unable to measure caller satisfaction. This would be a useful topic for further research. Finally, this study presents data from one institution, limiting the ability of our data to generate population-level inferences.

### Conclusions

In summary, our study describes the development and implementation of a COVID-19 patient triage hotline in a single health care system. The triage algorithm successfully diverted low-risk patients to suitable algorithm endpoints, while directing high-risk patients onward for immediate assessment. Data collected from hotline calls also enhanced our knowledge of typical symptoms and risk factors among community members, demonstrating that pandemic hotlines can aid in the clinical characterization of novel diseases. Although future innovation in the areas of triage algorithm design and remote work capabilities can certainly improve the operation of future pandemic hotlines, our work provides critical insight into the role that telephone-based triage systems play in facilitating health care delivery in times of crisis.

## Appendix

Multimedia Appendix 1 Supplemental material.
