# Supplementary material for: Utility of a Telephone Triage Hotline in Response to the COVID-19 Pandemic: Longitudinal Observational Study
Source: J Med Internet Res. 2021 Nov 1;23(11):e28105. doi: 10.2196/28105 (PMC8562418; doi:10.2196/28105)
Supplement: Multimedia Appendix 1 [file jmir_v23i11e28105_app1.docx]

**Supplementary Material**

**Supplemental Figure 1**

**Supp. Figure 1: Hourly call volumes on weekdays vs weekend days.** Distribution of hourly call volumes were similar on weekdays (orange) and weekend days (blue), although call volume was markedly greater on weekdays.

**Supplemental Figure 2**

**Supp. Figure 2: “Cough, Flu, and COVID-19-like Symptoms” e-visit questionnaire reported risk factors for adult and pediatric patients.** Percentages of risk factors reported by **(A)** adult (n=2132) and **(B)** pediatric (n=196) patients who completed the e-visit questionnaire within the study timeframe.
